# How is the theoretical domains framework applied to developing health behaviour interventions? A systematic search and narrative synthesis

**DOI:** 10.1186/s12889-019-7442-5

**Published:** 2019-08-28

**Authors:** Fiona Cowdell, Judith Dyson

**Affiliations:** 1 Faculty of Health, Education and Life Sciences, Birmingham City University, Birmingham, UK; 2 Faculty of Health Sciences, University of Hull, Hull, UK

**Keywords:** Health behaviour change, Theoretical domains framework (TDF), Capability, Opportunity, Motivation to behaviour (COM-B), Behaviour change wheel (BCW), Narrative review

## Abstract

**Background:**

Enabling behaviour change in health care is a complex process. Although the use of theory to inform behaviour change interventions is advocated, there is limited information about how this might best be achieved. There are multiple models of behaviour change, however, due to their complexity they can be inaccessible to both researchers and healthcare practitioners. To support health care practitioner behaviour change, this was addressed by the development of the Theoretical Domains Framework (TDF) in 2005. Citations of the TDF and associated papers have increased exponentially. Although not predicted or intended by the authors, the TDF has also been used to investigate health behaviour change interventions. Therefore our aim was to narratively synthesize empirical evidence on how the TDF and subsequent iterations have been applied in health behaviour change to inform future intervention development.

**Methods:**

Systematic search of four online databases, combined with searches for citations of key papers and key author searches, resulted in 3551 articles eligible for screening. Of these 10 met the pre-determined inclusion criteria. Screening of full-texts, data extraction and quality appraisal were independently performed by both authors. Disagreements regarding eligibility were resolved through discussion.

**Results:**

Of the 10 included studies three used the TDF and seven used subsequent iterations, the Capability, Opportunity, Motivation to Behaviour / Behaviour Change Wheel to assess and /or categorise behavioural determinants to identify relevant behaviour change techniques. Two studies reported feasibility testing. Most interventions were targeted at diet and exercise. Eight reported an explicit and systematic process in applying the framework.

**Conclusion:**

There is limited evidence of how the framework has been used to support health behaviour change interventions. In the included studies the process of using the framework is not always reported in detail or with clarity. More recent studies use a systematic and judicious process of framework application. From the limited evidence available we tentatively suggest that the steps proposed in the BCW appear to be sufficient for development of interventions that target health behaviour change interventions. Further research is needed to provide evidence in how the framework may be most effectively applied to intervention development.

**Protocol registration:**

PROSPERO CRD42018086896.

## Background

One important function of health care services is to support and encourage patients or the general population, at individual and community level, to adopt healthy behaviours to reduce the risk of ill-health, maintain health and self-manage long-term conditions [1–3]. However, health behaviour change is a complex process. Although the Medical Research Council guidelines for complex interventions [4] and National Institute for Health and Care Excellence (NICE) recommendations [1, 2] advocate the use of theory to inform health behaviour change interventions there is limited information about how this might best be achieved. Systematic reviews of existing evidence demonstrate the effectiveness of such an approach [5, 6]. The explicit use of theory allows us to understand the mechanisms of change in behaviour and to replicate interventions [7]. There are multiple models of behaviour change that have been used in healthcare (e.g. the Theory of Reasoned Action [8], the Theory Planned Behaviour [9] and the Transtheoretical Model of Behaviour Change [10]). However due to their complexity they can be inaccessible to both researchers and healthcare practitioners.

Michie and colleagues addressed these challenges for the field of implementation science (supporting health care practitioner behaviour change) by using a consensus approach to develop the Theoretical Domains Framework (TDF) [11]. This brings together 33 models of behaviour or behaviour change and includes 128 separate constructs [11]. The TDF has 11 theoretical domains that explain the potential determinants of behaviour (*knowledge, skills, social/professional role and identity, beliefs about capabilities, beliefs about consequences, motivation and goals, memory attention and decision processes, environmental context and resources, social influences, emotion and action planning).* Subsequent development of the TDF led to validation [12] with 14 domains where *optimism, reinforcement* and *intentions* were identified as important and added (rather than being embedded in the earlier 11). Latterly, the Behaviour Change Wheel (BCW) [13] was developed as a “behaviour system”, designed to link from identification of determinants of behaviour (using the TDF) to the mapping of appropriate behaviour change techniques (BCTs) to inform interventions.

It consists of “COM-B” (Capability, Opportunity and Motivation to Behaviour) at the hub of the wheel. Use of the COM-B helps identify domains of the TDF most likely to influence behaviour change. In practice, domains of the TDF have been mapped to the COM-B. For example, “Capability” includes the domains *knowledge* and *skills*, “Opportunity” incudes *social influences* and *environmental context/resources* and “Motivation” includes *beliefs about capabilities* and *emotion* [13]. The hub (COM-B) of the BCW is encircled by nine intervention functions (*education, persuasion, incentivisation, coercion, training, restriction, environmental restructuring, modelling and enablement*) and the outer of the three rings seven policy categories (*communication, guidelines, fiscal, regulation, legislation, environmental/social planning and service provision*). The TDF and BCW (including COM-B) provide a comprehensive eight stage process to intervention design recommended by the authors of the framework: i) define the problem, ii) select the target behaviour, iii) specify the target behaviour and identify iv) what needs to change, v) intervention functions, vi) policy categories, vii) behaviour change techniques (BCTs) and viii) mode of delivery [13].

It is thirteen years since the publication of the TDF and there is limited definitive instruction on how to apply it in intervention design and testing. Michie and colleagues [7] demonstrate how to link behavioural determinants to BCTs. Taylor and colleagues [14] offer a worked example of applying the TDF to healthcare practitioner behaviour. More recently a guide on how to design BCT based interventions has been published [15]. Since 2012 citations of the TDF and associated papers has increased exponentially. Although not predicted or intended by the authors, the TDF and subsequent iterations has also been used to investigate health behaviour change interventions.

The objective of this review was to identify and narratively synthesise papers in which the TDF, or subsequent iterations (hereafter referred to as “the framework”), have been used in relation to health behaviour change interventions with a specific focus on those which report on intervention development and/or testing to inform optimal use in future studies.

## Methods

### Search strategies and selection criteria

The electronic databases Cumulative Index to Nursing and Allied Health Literature (CINAHL), Medline, PsychINFO and Cochrane were searched using the key terms

“theoretical domains framework” or TDF or COM-B or “behav* change wheel” or BCW

NOT

Implement* or improv* or quality or guideline* or EBP or "evidence based practice".

Two further searches were conducted using Google Scholar i) citations of key papers [11, 12, 15] and ii) key author searches for papers from Charles Abraham, Lou Atkin, James Cane, Jill Francis, Marie Johnston, Rebecca Lawton, and Robert West. The rationale for the latter was that the framework was first cited as the “Theoretical Domains Framework” in 2009 so papers prior to this may not otherwise have been identified and these authors are recognised experts in the field. The search was undertaken in August 2018. Inclusion and exclusion criteria are summarised in Table 1.

**Table 1:** Inclusion and exclusion criteria

Inclusion	Exclusion
Published from 2005 (original publication of the TDF) onwards	
Published in English language	Published in languages other than English (as there were no resources for translation)
Papers focusing on health behaviour	Papers focusing on healthcare practitioner behaviours
Empirical papers that report design and/ or testing of interventions underpinned by the TDF or subsequent iterations of the framework	

Title screening was conducted by a research associate and FC independently. Abstracts were screened for eligibility by JD and FC in accordance with the inclusion and exclusion criteria. Full texts were obtained where there was any doubt about eligibility and authors were contacted in cases of uncertainty. At each stage disagreements were discussed to resolution.

### Analysis

The focus of our review is empirical studies that engaged in intervention design and testing, which we narratively synthesised following the approach of Ferrari [16]. This offers a systematic but straightforward approach appropriate to the nature and homogeneity of the included papers. Data were extracted using a bespoke data extraction table in which we recorded: study design, target group, health behaviour, intervention and framework use. We grouped papers according to the targeted health behaviour. Each paper within the group was discussed and evaluated and application of the framework summarised. Main points were synthesised in relation to our review question and underpin suggestions for future research. Quality of intervention reporting was assessed according to the Template for Intervention Description and Replication (TIDieR) Checklist [17] completed by both authors.

## Results

### Search results

**Fig. 1:**
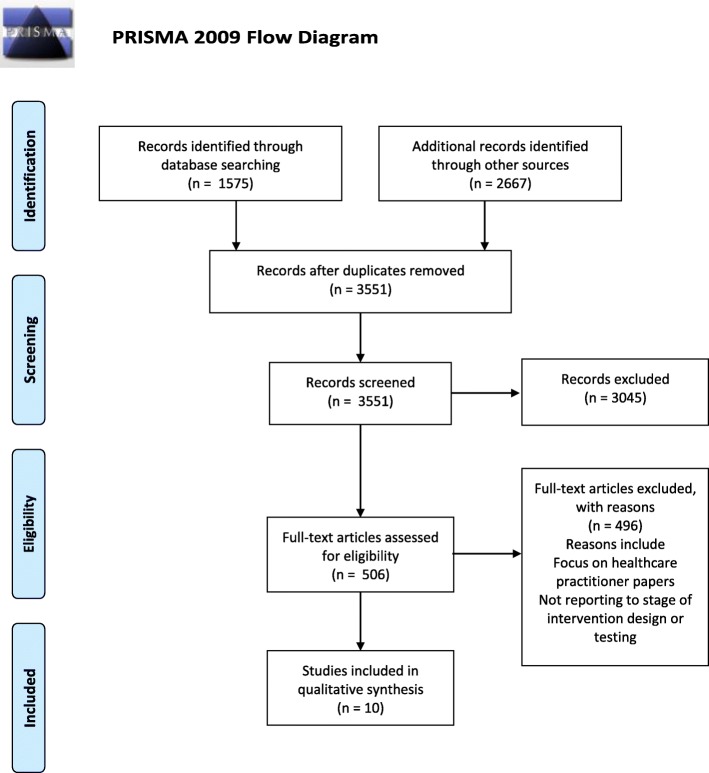
PRISMA Flow Diagram

From the original 3551 papers identified 10 met the inclusion criteria. The search process is summarized in Fig. 1 (Preferred Reporting Items for Systematic Reviews and Meta-Analyses: PRISMA flow chart [18]). A summary of included papers is provided in Table 2 and the quality of intervention reporting (design and where applicable delivery and evaluation) is reported in Table 3 [17]. A summary of the key points of the papers is provided followed by a narrative review [16] of papers according to intervention focus.

**Table 2:** Description of included papers and use of framework (*n* = 10)

First author, year (ref)	Study design/method	Target group	Health Behaviour	Intervention	Framework use
Curtis, 2015 UK [19]	BCW framework with user-centered design informed app intervention development process. Existing evidence, supplemented by thematic analysis of data from focus groups (*n* = 9) with weight management case workers and parents of children aged 5–11 years (*n* = 46) and experts.	Parents	Provision of appropriate food portion sizes	A user-centred healthy eating app app to target childhood weight management	COM-B used to assess determinants through consideration of current evidence, focus groups and consultation with experts. BCW used to map relevant BCTs
Mann, 2014USA [20]	Intervention development by the research team (no participants).	People with hypertension	Lifestyle including a healthy diet (including reduced sodium intake) and exercise	An m-Health version of the existing DASH (Dietary Approaches to Stop Hypertension) intervention. A hypertension reduction lifestyle modification system	Implicit use of COM-B to identify behaviour change techniques
Martin 2015 Ireland [21]	Intervention development and trial design research team design no participants. Cluster RCT	Children 8–11 years	Exercise	“Active Classrooms” 8 week classroom based physical intervention aimed to increase physical activity	Barriers from the literature categorised to COM-B and BCW used to identify BCTs
McEachan, 2016 UK [22]	Feasibility RCT (*n* = 120) of an existing intervention. Consenting women randomly allocated to HAPPY or usual care. Outcome measures for full trial explored.	Overweight or obese women during and after pregnancy	Make healthy food choices and increase physical activity	“HAPPY” Healthy and Active Parenting Programme for early Years aimed at reducing risk of obesity in infants of overweight or obese women. (Details of intervention in [23])	Interventions were mapped to behavioural determinants which were categorised to the TDF
Munir 2018 UK [24]	Intervention development involving focus group with NHS staff (*n* = 39) to identify barriers and facilitators. Data used with taxonomy of Behaviour Change Techniques to identify strategies for behaviour change. Participant sub-group tested several electronic self-monitoring devices.	Sedentary office workers	Reduction in time spent sitting	“Stand More AT Work (SMArT Work)”. Four devices that monitor and feedback on sitting/inactivity.	Intervention design guided by the BCW eight stage process.
Robinson, 2013 UK [25]	Intervention development and feasibility testing in 4 week trial involving overweight and obese university staff (*n* = 12). Semi-structured interviews to assess acceptability and uncover barriers to use. Adherence monitored electronically	Overweight people	Attentive eating	A smartphone based attentive eating intervention to reduce calorie intake	Intervention design guided by BCW eight stage process.
Taylor, 2013 UK [26]	Intervention mapping framework used i. Needs assessment and review of evidence base ii. Desired outcomes and barriers to these identified and mapped in interviews (*n* = 12), focus groups (*n* = 27) and surveys with parents and grandparents (*n* = 1242) and health care practitioners (*n* = 20). Barriers mapped according to psychological determinants. iii.theory based methods for overcoming barriers identified iv. design of intervention v adoption and implementation in Children Centres	Overweight or obese women during and after pregnancy	Make healthy food choices and increase physical activity	“HAPPY” Healthy and Active Parenting Programme for early Years to prevent childhood obesity	TDF used to needs assess, identification of barriers, mapping to BCTs.
Tombor 2016 UK [23]	Intervention development in comprising three main stages i. preparation, involving focus groups healthcare providers and interviews with pregnant smokers to establish what would need to change in pregnant smokers or the environment ii. design iii. Piloting with non-pregnant users (*n* = 6)	Pregnant smokers	Smoking cessation	“SmokeFree Baby” smartphone app to help pregnant women stop smoking. Includes brief advice, motivational messages, positive role models, information about foetal development and a video diary.	BCW/COM-B to guide interviews and focus groups and to select BCTs. The BCW eight step process was followed.
van Agteren 2018 Australia [27]	Intervention development using existing evidence base, interviews (*n* = 16) and focus groups (*n* = 5) with smokers and health professionals to assess needs	Smokers	Smoking cessation	“Kick.it” a mobile health intervention involving a logging smoking and cravings, reminders, social network, educational and motivation videos.	TDF to conduct a needs assessment mapped to BCTs which underpinned the intervention
Webster, 2015 UK [28]	Intervention development involved review of existing evidence, interviews with male clinic attendants (*n* = 20) followed by a workshop of experts (*n* = 13). Three focus groups (*n* = 16) and interviews (*n* = 7) with clinic users. Intervention designed to address target behaviours. User testing (*n* = 16) to refine intervention.	Heterosexual men	To increase condom use	“MenSS” (Men’s Safer Sex), an interactive digital intervention to prevent sexually transmitted infections	BCW to categorise behavioural determinants (from literature, experts and interviews with target population) and to select BCTs

**Table 3:** Quality of intervention reporting in included papers

	Rationale stated?	Materials described?	Procedure described?	Expertise/ background of person delivering? (planned)	Mode of delivery reported? (planned)	Location? (Planned)	When and how much? (planned)	Tailoring? (planned)	Modifications?	Intervention fidelity? (planned)	Intervention fidelity? (assessed as planned)
Curtis et al. 2015 [19]	✓	✓	✓	n/a	✓	✓	n/a	✓	✓		n/a
Mann et al. 2014 [20]	✓	✓	✓	n/a	✓	✓	✓	Not reported	Not reported	✓	n/a
Martin and Murtagh 2015 [21]	✓	✓	Examples offered	✓	✓	✓	Not reported	Not reported	Not reported	✓	n/a
McEachan et al. 2016 [22]	✓	✓	✓	Reported elsewhere [26]	✓	✓	✓	✓	Reported elsewhere [26]	✓	✓
Munir et al. 2016 [24]	✓	✓	✓	n/a	✓	✓	n/a	✓	✓	✓	✓
Robinson et al. 2013 [25]	✓	✓	✓	n/a	✓	✓	n/a	✓	✓	✓	✓
Taylor et al. 2013 [26]	✓	✓	✓	✓	✓	✓	✓	✓	✓	✓	n/a
Tombor et al. 2016 [23]	✓	✓	✓	n/a	✓	✓	n/a	✓	✓	✓	✓
Van Agteren 2018 [27]	✓	✓	✓	n/a	✓	✓	✓	✓	✓	✓	✓
Webster et al. 2015 [28]	✓	✓	Reported elsewhere [29]	n/a	✓	✓	n/a	✓	✓	✓	n/a

*n/a = non applicable

Included papers were published in 2013 onward and conducted in the UK [19, 22–26, 28], Ireland [21], Australia [27] and the USA [20]. The TDF was used in three papers [22, 26, 27], and the Com-B/BCW in seven [19–21, 23–25, 28]. The framework was used solely to identify relevant BCTs [20] or to assess and/or to categorise behavioural determinants or barriers and to identify relevant BCTs or to both [19, 21–23, 27, 28]. The eight stage BCW process was used in three studies [23–25]. In one case a framework based intervention, reported elsewhere [26] was feasibility tested [22].

The majority of interventions were technology based [19, 20, 23–25, 27, 28], of these, one included direct contact with a health care provider [20], fewer were face to face delivery only [21, 22, 26]. Interventions targeted children and young people [21] parents [19], overweight pregnant women [22, 26], pregnant smokers [23], smokers [27], sedentary office workers [24], overweight people [25], heterosexual men [28] and people with hypertension [20]. Interventions were designed to target sexual health/contraception [28], smoking [23, 27], diet and exercise [19, 21, 22, 24–26] and specific health condition related behaviours [20]. Each of these categories are addressed in turn below with a specific focus on application of the framework.

### Health behaviours targeted

#### Sexual health and contraception

There was one UK based study that developed an intervention to address sexual health and contraception, this was predominantly male focused [28]. Using the BCW/Com-B throughout, Webster and colleagues [28] clearly specified the target behaviour and investigated the barriers to condom use through a literature review and interviews with the target population. Interviews also established potential intervention design, content and mode of delivery. Two workshops with experts (one before and one after interviews) involved mapping barriers to explanatory domains, considering intervention functions and design of intervention content. User testing and focus groups refined the ultimate “MenSS” intervention design which was feasibility tested and evaluated.

#### Smoking

There were two studies in this category, one conducted in the UK [23] and one in Australia [27]. Tombor [23] developed “SmokeFree Baby” a smart phone app for pregnant smokers and van Agteren [27] developed the Kick.it mobile health intervention designed to support smoking cessation. Tombor [23] used the TDF to underpin focus groups of healthcare providers and interviews with pregnant smokers to establish what would need to change in pregnant smokers or the environment and conducted a pilot test of the app. Van Agteren [27] used interviews and focus groups, underpinned by the TDF, with smokers and healthcare professionals to assess needs which were mapped to BCTs which and used to inform intervention design.

#### Diet and exercise

There were six studies in this category. Five were conducted in the UK [19, 22, 24–26] and one in Ireland [21]. Two focused on the “HAPPY” intervention [22, 26] which was designed for overweight or obese women during and after pregnancy. One focused on children [21], one the parents of children [19], one on overweight people [25] and one sedentary office workers [24]. Two interventions were apps [19, 25], one was a monitoring and feedback device [24] and three were designed to be delivered face to face [21, 22, 26]. Taylor and colleagues [26] conducted literature reviews to establish the needs and theoretical determinants to pregnant women adopting a healthy diet and exercise regime. The literature data were supplemented by interviews, focus groups and surveys of parents, grandparents and healthcare professionals. The determinants to diet and exercise behaviours were categorised to the TDF and subsequently mapped to relevant BCTs listed within an existing taxonomy [30]. BCTs underpinned the development of a programme plan. Implementation and evaluation plans were also developed. The intervention itself was subsequently tested by McEachan and colleagues [22] in a feasibility RCT with babies’ weight as the primary outcome measure. The intervention was evaluated to be acceptable, feasible and demonstrated promising results for infant obesity prevention. The theoretical underpinning of the intervention was not reported or discussed in this paper. Curtis and colleagues [19] used a three stage approach to designing an m-health app targeted at parents to support childhood weight management. A literature review combined with focus groups with stakeholders (case workers and parents) led to selection and definition of the target behaviour, “providing appropriate food portions”. COM-B, TDF and existing evidence was used to underpin the focus group question schedule to explore barriers, facilitators and preferences for the final intervention. Barriers categorised to the TDF were mapped to BCTs through use of the BCW. Although the authors report piloting the resulting intervention the results of this are not presented.

Robinson and colleagues [25] reviewed the literature to identify a target behaviour, “eating attentively”, as a means of reducing calorie intake and aiding weight loss. They used the COM-B to list strengths of smartphone technology. The authors report the app content but make no further reference to the framework. Feasibility testing with obese adults was conducted. Primary outcome measures were i) frequency of use, ii) qualitative evaluation of the effects of using the app’ and factors affecting use and iii) self-reported acceptance. Whilst not a primary outcome measure weight changes were monitored. The intervention evaluation demonstrated equivocal results on all measures. Munir et al. [24] comprehensively applied the eight stages of the BCW. Martin and Murtagh [21] described intervention design and present a protocol for a cluster RCT to test a classroom based 8 week intervention to increase activity. A literature review was used to identify barriers and facilitators to integrating physical movement into classroom activities. These were categorised to the COM-B and the BCW used to identify appropriate BCTs.

#### Specific behaviours for specific groups

One study was included in this category, a US based, m-health intervention addressing dietary approaches to the management of hypertension [20]. Mann and colleagues [20] considered diet and exercise focusing on specific blood-pressure related elements (e.g. salt intake) and therefore we have categorised it as an intervention for a specific health condition. These authors adapted the mode of delivery of the effective and established “DASH” (Dietary Approaches to Stop Hypertension) intervention from face to face and web-based to mobile app. Mann and colleagues [20] cite the COM-B/BCW and appear to include three BCTs; educational clips, coaching and motivational interviewing. There is no further description of the use of theory in the development of the M-health “DASH” intervention.

Above we have presented our findings with regard to our review question “How is the Theoretical Domains Framework applied in health behaviour change interventions?”. Although there is no established “gold standard” here we critique the process of applying the framework in the light of published guidance and examples [4, 5, 31, 32]. Eight of the included papers reported an explicit and systematic process in applying the framework to intervention design and testing [19, 21–24, 26–28].

All defined the target health behaviour and gave a clear account of relevant behavioural determinants which were established through a range of techniques. Although the use of theory in intervention design was thoroughly reported by Curtis et al., the description of the process was relatively complex to follow. Two papers [20, 25] were less explicit in their application of the framework. Robinson and colleagues [25] “*assume*(d)”the barriers and facilitators to eating attentively rather than systematically investigating these. Although the authors state they used the BCW to understand the behavioural determinants to the target behaviour in fact they appear to have used it to understand the determinants to intervention uptake. Whilst the electronic process of the m-health DASH intervention is explicit and detailed, Mann and colleagues [20] make limited reference to the contribution of behaviour change theory. The most recent three papers offer the clearest and most detailed explanation of application of the framework to intervention design [23, 24, 27].

## Discussion

The aim of our review was to establish how the TDF and subsequent iterations of the framework have been applied in health behaviour change interventions. Following a rigorous selection process, 10 papers met our inclusion criteria. All ten reported intervention development and two of these went on to test the feasibility of the intervention. The TDF was used in three papers and the COM-B/BCW in seven. Seven interventions were predominantly technology based and three were face to face. Interventions were categorised according to target health behaviours which were sexual health/contraception, smoking cessation, diet and exercise and specific behaviours for specific groups.

Critique of the framework in the included papers suggested that it was time consuming to apply (particularly where there are multiple target behaviours) [28] and requiring intervention developers to have a knowledge of both the process and relevant BCTs [28]. Webster and colleagues [28] report the framework enables a clear process of design and makes explicit active ingredients (BCTs) which allows intervention replication.

There are two other reviews that have considered the use of the TDF. A synthesis of the use of the TDF in 2012 identified 133 papers which cite the framework, 21 of these were empirical studies and of these only four investigated the health behaviour change interventions [33]. Only one of these papers involved intervention design and is therefore also included in our review. Birken et al. 2017 [34] sought to elicit the rational of authors in combining the Consolidated Framework for Implementation Research (CFIR) and the TDF. All of their 12 included papers focused on practitioner rather than patient and general public health behaviours. There is one review protocol [35] considering how the framework is applied in designing interventions to support healthcare practitioner behaviour change.

Although we were systematic in our search, it is possible that we have not identified papers published before common use of the name “Theoretical Domains Framework (TDF)” which appears to have first been documented in 2009 [36]. Whilst we acknowledge that the TDF was designed to support healthcare practitioner behaviours it has also been extensively cited in the health behaviour change literature thus justifying this review. This number of citations may be due to the lack of an alternative framework. The only comparator we are aware of is Fishbein’s approach [8] which was developed with specific regard to people with Human Immunodeficiency Virus. However despite many citations in empirical papers only twelve use the TDF and subsequent iterations for intervention design and testing.

## Conclusion

There is limited evidence of how the framework has been used to support health behaviour change interventions. In the included studies the process of using the framework is not always reported in detail or with clarity. The more recent studies use a systematic and judicious process of framework application. Due to small numbers and unclear reporting of the use of the framework in two of the included papers is not possible to comment on any association between the use of robust methods for intervention development and feasibility or effectiveness of the resulting intervention; this is worthy of consideration in future reviews. From the limited evidence available we tentatively suggest that the steps proposed in the BCW appear to be sufficient for development of interventions that target health behaviour change interventions. Further research is needed to provide evidence in how the framework may be most effectively applied to intervention development.

## Data Availability

Not applicable

## Abbreviations

BCTs: behaviour change techniques
BCW: Behaviour Change Wheel
COM-B: Capability, Opportunity, Motivation to Behaviour
NICE: National Institute for Health and Care Excellence
TDF: Theoretical Domains Framework
TIDieR: Template for Intervention Description and Replication
